# Unraveling Wing Shape Variation in Malaria Mosquitoes from the Arctic Edge: A Geometric Morphometric Study in Western Siberia

**DOI:** 10.3390/ani15202949

**Published:** 2025-10-11

**Authors:** Ximena Calderon, Gleb Artemov, Vladimir A. Burlak, Svetlana Alexeeva, Raquel Hernández-P, Manuel J. Suazo, Laura M. Pérez, Hugo A. Benítez, Margarita Correa

**Affiliations:** 1 Laboratory of Evolutionary Cytogenetics, Department of Genetics and Cell Biology, Tomsk State University, Tomsk 634050, Russia; ximena.calderon0227@gmail.com (X.C.); g-artemov@mail.ru (G.A.); flywings@mail.ru (V.A.B.); sveta.alexx@mail.ru (S.A.); 2 Departamento de Ecología Evolutiva, Instituto de Ecología, Universidad Nacional Autónoma de México, Ciudad de Mexico 04510, Mexico; jraquel.hdz@gmail.com; 3 Vicerrectoría de Investigación y Postgrado, Universidad de La Serena, La Serena 1700000, Chile; suazo.mj@gmail.com; 4 Departamento de Ingeniería Industrial y de Sistemas, Universidad de Tarapacá, Arica 1000000, Chile; lperez@uta.cl; 5 Laboratorio de Ecología y Morfometría Evolutiva, Instituto One Health, Facultad de Ciencias de la Vida, Universidad Andrés Bello, República 440, Santiago 8370134, Chile; 6 Millennium Institute Biodiversity of Antarctic and Subantarctic Ecosystems (BASE), Santiago 7800003, Chile; 7 Cape Horn International Center (CHIC), Centro Universitario Cabo de Hornos, Universidad de Magallanes, Puerto Williams 6350000, Chile; 8 Centro de Investigación de Estudios Avanzados del Maule, Universidad Católica del Maule, Talca 3466706, Chile

**Keywords:** malaria, shape analysis, geometric morphometrics, hybrids, *Anopheles*

## Abstract

**Simple Summary:**

In Western Siberia, the *Anopheles maculipennis* subgroup includes three malaria vectors *An. messeae*, *An. daciae*, and *An. beklemishevi*, and their hybrids, which are difficult to tell apart using traditional traits such as egg exochorion. We combined molecular identification with geometric morphometrics of wing venation to evaluate differences in wing shape and size among species. We found statistically significant differences in wing shape and centroid size, with hybrids showing intermediate morphology and overlap with their parental species. Landmarks on the radial and medial veins contributed most to species separation. We did not evaluate environmental adaptation, and wing shape differences were not explained by size. These findings support the use of wing morphometrics, together with genetic data, to improve species delimitation and surveillance of malaria vectors in temperate regions.

**Abstract:**

In Russia, Western Siberia, *Anopheles* from *maculipennis* subgroup comprises three vector species: *An. messeae*, *An. daciae*, *An. beklemishevi*, and the hybrid between *An. messeae* and *An. daciae* (*Anopheles* m-d), which exhibit complex cryptic morphological traits. Traditional morphological methods, such as egg morphology and exochorion coloration, have proven insufficient for reliably distinguishing these closely related species due to overlapping characteristics and high intra-species variability. To overcome these limitations, geometric morphometrics (GM) has emerged as a powerful tool for analyzing cryptic morphology. This article focuses on wing venation patterns, where GM provides precise, quantitative data based on defined anatomical landmarks, enabling detailed assessment of size and shape variation among species. Procrustes ANOVA, principal component analysis (PCA), and canonical variate analysis (CVA) were employed to assess shape variation and species differentiation. Centroid size and its relationship to shape variation were examined using multivariate regression. Despite significant morphological differences, the overlap observed in hybrids (*An.* m-d) reflects their intermediate position between the parental species. Our analyses revealed significant differences in wing shape and size among *An. messeae*, *An. daciae*, *An. beklemishevi*, and their hybrids, with hybrids showing intermediate morphologies. Landmarks on radial and medial veins were the most consistent contributors to species separation. No evidence of static allometry was detected, and wing shape differences were not explained by size. These findings demonstrate that wing morphometrics, combined with molecular identification, provides a reliable framework for species delimitation and surveillance of malaria vectors in temperate regions.

## 1. Introduction

Malaria remains a significant public health problem caused by unicellular protozoan parasites of the genus Plasmodium and transmitted by *Anopheles* mosquitoes. According to the World Health Organization (WHO) World Malaria Report 2023, there were an estimated 249 million malaria cases globally in 2022, resulting in 608,000 deaths [1].

The mosquitoes of the *maculipennis* subgroup have a Palearctic distribution, with three species of malaria mosquitoes identified in Western Siberia: *An. messeae*, *An. daciae*, *An. beklemishevi* [2,3], and *An. claviger* [4].

Despite of that the most species of *maculipennis* subgroup can be differentiate by egg morphology and the coloration of exochorion, some of them can be differentiated by cytogenetic characteristics, polythene chromosome banding patterns [5] as well as heterochromatin features in ovarian nurse cells [6,7]. A cryptic species *An. daciae* has been delineated from *An. messeae* based on specific chromosome inversions composition [8], taxon print analysis [9], ITS2 sequences and exochorion coloration [10] as well as ecological features [8]. Recently, using ITS2 sequences as diagnostic markers hybrids between *An. messea* and *An. daciae* (*An.* m-d) was found in some locations of European [11] and Western Siberian part of Russia and Kazakhstan [3].

While traditional morphological characters for distinguishing these cryptic species can be limited and potentially confusing, geometric morphometric analysis of wing venation has proven valuable in providing well-defined landmarks and precise quantitative information for species identification [12,13,14]. This method, employed in conjunction with genetic approaches, allows for a more comprehensive understanding of the morphological variations within the *maculipennis* subgroup. Geometric morphometrics also provides a powerful framework to study size and shape variation independently, capturing both heritable components and environmentally induced variation [15]. Recent developments have expanded their applications in ecology and evolutionary biology [16], and in mosquitoes, wing venation landmarks have been shown to be reliable for assessing population structure and phenotypic variability [17].

Geometric morphometrics has been increasingly applied to *Anopheles* species, providing insights into taxonomy and ecology. Jeon et al. [18] combined wing morphometrics with molecular phylogeny to differentiate eight *Anopheles* species in Korea, showing the difficulty of separating cryptic taxa. In Italy, Bellin et al. [19] applied morphometrics with machine learning to the *maculipennis* complex and achieved up to 83% classification accuracy. Ayala et al. [20] studied *An. funestus* in Cameroon and demonstrated that wing variation is influenced by chromosomal and environmental factors, while Gómez et al. [21] reported environmentally associated wing shape differences in *An. albimanus* in Colombia. Despite these advances, very few studies have applied geometric morphometrics to *Anopheles* species, and none have yet examined *An. messeae*, *An. daciae*, *An. beklemishevi*, and their hybrids in Eurasia.

Understanding morphological variation, particularly in wing traits, can provide insights into species differentiation and dispersal patterns, which may ultimately influence the epidemiology of mosquito-borne diseases [22,23]. Therefore, this study aimed to investigate the variation in wing size and shape among *An. messeae*, *An. daciae*, *An. beklemishevi*, and their hybrids across contrasting environments in Western Siberia. Our focus was to evaluate morphological differentiation using geometric morphometrics in combination with molecular identification, in order to strengthen species delimitation within the *maculipennis* subgroup.

## 2. Materials and Methods

### 2.1. Study Area and Sampling Sites

**Figure 1:**
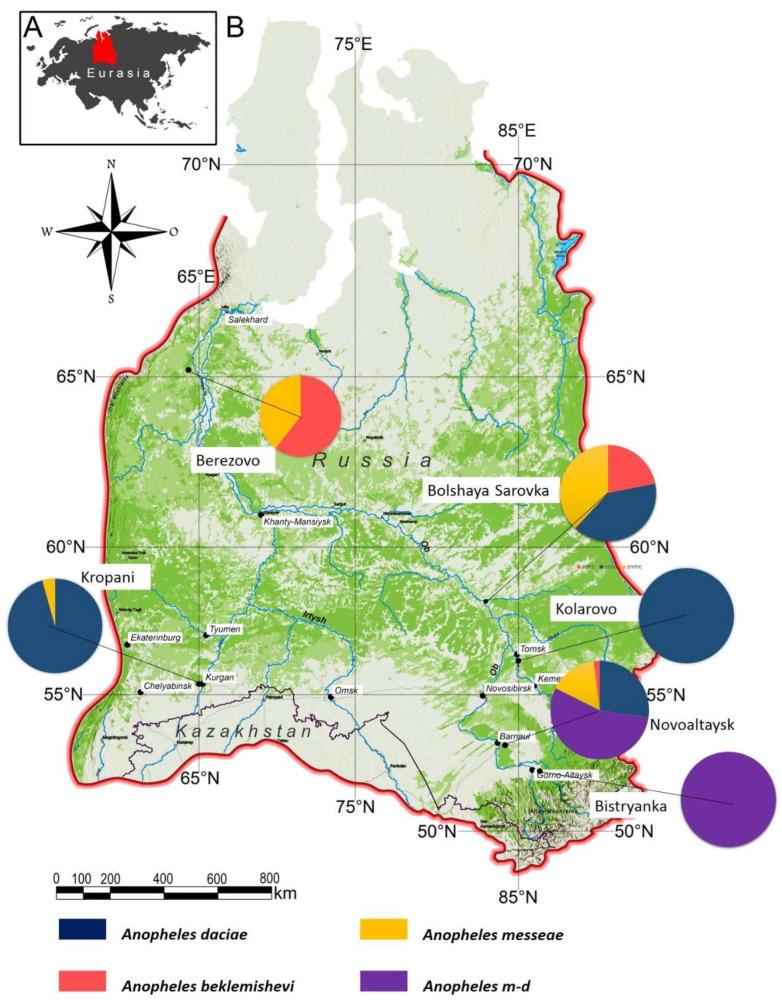
Map of the six mosquito collection sites in Western Siberia included in this study. Pie charts indicate the relative proportions of *An. messeae*, *An. daciae*, their hybrids (*An.* m-d), and *An. beklemishevi* at each site.

Mosquito specimens of the *Anopheles maculipennis* complex were collected from six ecologically distinct localities across Western Siberia, Russia, during the mosquito breeding season of 2021. Western Siberia was chosen because it represents the northern distribution limit of the *maculipennis* subgroup, where contrasting ecological conditions (from taiga to steppe) overlap with the presence of *An. messeae*, *An. daciae*, *An. beklemishevi*, and their hybrids. This region thus provides a natural setting to assess morphological variation across environmental gradients and hybrid zones. The selected sites represent a broad latitudinal and ecological gradient, ranging from northern taiga (Berezovo) to southern forest-steppe (Kropani, Novoaltaysk) and foothill forest zones (Bystryanka) (Figure 1). These environments differ in temperature, altitude, precipitation, and vegetation, providing a diverse ecological context for the sampling sites (see Table 1 and Table 2).

A total of 299 adult female specimens were selected from a larger collection > 6700 *Anopheles* mosquitoes collected across the six study sites for geometric morphometric analysis. The subset was chosen to ensure broad representation across localities and species within each species–locality group, specimens were selected at random, and only individuals with intact wings were included to avoid measurement bias.

Specimens were preserved in 96% ethanol and stored at −21 °C. Species identification was performed using molecular techniques based on the internal transcribed spacer 2 (ITS2) region, applying the PCR-RFLP protocol described by Artemov et al. (2021) [24]. 202In brief, ITS2 was amplified using primers 5,8S_vdir (5′-TGTGAACTGCAGGACACATG-3′) and 28S (5′-ATGCTTAAATTTAGGGGGTA-3′). PCR products were digested with the restriction enzyme RsaI, allowing differentiation of *An. messeae* and *An. daciae* based on restriction fragment patterns, while *An. beklemishevi* was distinguished by the length of its PCR product. Full details of the reagents and conditions are provided in Supplementary Table S1. Among the selected specimens, 128 were identified as *An. daciae*, 64 as *An. messeae*, 48 as *An. beklemishevi*, and 59 as *An. messeae* × *An. daciae* hybrids.

Environmental parameters were obtained for each site to contextualize wing shape variation. These included the start and end of the breeding season, mean seasonal temperature, total precipitation, precipitation rate (mm/day), sum of effective temperatures (SET), and the number of theoretical (Tgtz), realized (Fgtz), and effective (Egtz) gonotrophic cycles. Meteorological data were obtained from the environmental data were obtained from the Russian open-access source “Pogoda i Klimat” (https://pogodaiklimat.ru) (accessed on 13 January 2025) [25], and gonotrophic cycles were calculated following Yakimenko et al. (2013) [26], using the formula ∑(Tmean − 9.9 °C) = 36.5 × days.

### 2.2. Wing Preparation and Landmark Digitization

**Figure 2:**
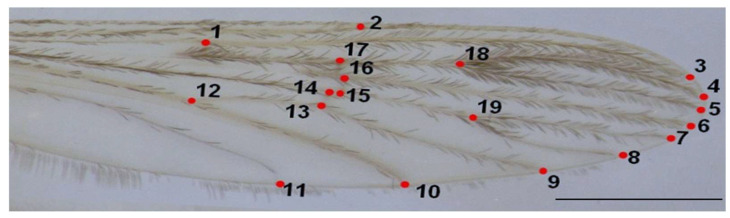
Right wing of *An. messeae*. Numbers indicate the positions of the 19 landmarks used in the geometric morphometric analysis. Scale bar = 1 mm.

The right wing of each mosquito was dissected for analysis, dehydrated in two baths of 100% ethanol (10 min each), mounted in Euparal on a slide, and photographed using an Olympus SZX9 stereo-microscope with SZ61 camera at 20× magnification (Olympus, Santiago, Chile). A 1 mm scale was included for calibration (Figure 2).

Nineteen homologous landmarks were digitized per wing using TpsDig2 v2.31 [27], focusing on intersections of wing veins. Landmark configurations were aligned using Generalized Procrustes Analysis (GPA) to remove non-shape variation such as size, position, and rotation [28]. Each wing was digitized twice to assess consistency, and a Procrustes ANOVA was used to evaluate measurement error, confirming that individual variation exceeded error.

### 2.3. Geometric Morphometric and Statistical Analysis

**Figure 3:**
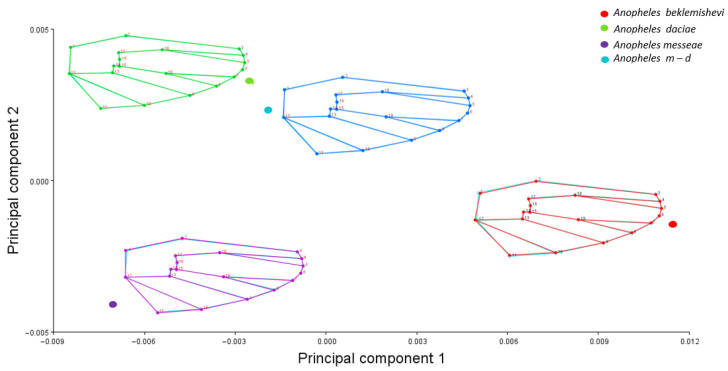
Principal Component Analysis (PCA) of consensus wing shapes of *An. messeae*, *An. daciae*, *An. beklemishevi*, and their hybrids. Wireframes represent average landmark configurations obtained after Procrustes superimposition for each group.

Wing shape variation was explored using Principal Component Analysis (PCA) based on the covariance matrix of Procrustes-aligned coordinates. As a preliminary step, we visualized the consensus wing shapes of each group by calculating average landmark configurations and projecting them in a PCA (Figure 3). This graphical representation allowed us to illustrate the typical wing shape of each species before conducting the canonical variate analysis (CVA). Although this approach is descriptive and not inferential, it helps to visualize interspecific differences in wing morphology. Canonical Variate Analysis (CVA) was performed to assess separation among species, with statistical significance evaluated through 10,000 permutations using Mahalanobis distances.

Wing size was estimated by calculating centroid size (CS), defined as the square root of the sum of squared distances from each landmark to the centroid. Procrustes ANOVA was used to test for size differences between species, with significance determined by 10,000 non-parametric permutations (*p* < 0.001). Results were visualized using violin plots.

Static allometry was evaluated through multivariate regression of wing shape (Procrustes coordinates) on centroid size. Regression scores were plotted in tangent space to visualize the allometric component of shape. All analyses were performed using MorphoJ v1.08a [22], and the R packages geomorph [29] and ggplot2 [30] within RStudio (2025.09.1+401) [31].

## 3. Results

For interspecific comparisons, individuals were pooled by species across all localities. This approach allowed us to focus on species-level differentiation, rather than environmental or geographical effects; therefore, we did not attempt to estimate the influence of environmental factors on wing morphology in this framework.

### 3.1. Geographical Distribution of Anopheles Species

The survey across six ecologically diverse sites in Western Siberia revealed marked differences in species composition and relative abundance (Figure 1). *Anopheles daciae* dominated in the southern sites of Kropani and Kolarovo, while hybrid forms (*An. messeae* × *An. daciae*) were prevalent in Novoaltaysk and Bystryanka. In the northern locality of Berezovo, An. *beklemishevi* co-occurred with *An. messeae*, the former being dominant. Bolshaya Sarovka showed similar proportions of each species of *An. daciae*, *An. messeae*, and *An. beklemishevi*, highlighting its role as a transition zone in species distribution.

### 3.2. Statistical Analysis

A total of 299 specimens were analyzed using geometric morphometric techniques. Procrustes ANOVA revealed that the mean square for individual variation exceeded measurement error, confirming the consistency of landmark digitization (Table 3).

The PCA showed that most of the shape variation was explained in the first three dimensions, accounting for 42.9% (PC1 = 20.3%; PC2 = 15%; PC3 = 11.51%) of the total shape variation between species was superimposed to analyze interspecific shape variation. To identify statistical differences, a Procrustes ANOVA was performed using species and locality as factors, revealing significant differences in both centroid size and wing shape (Table 4).

The averaged PCA (Figure 3) demonstrated clear wing shape differentiation within the morphospace, with *An.* m-d showing similarity to *An. daciae*, while *An. messeae* exhibited an opposite distribution along the PC1 axis relative to *An. beklemishevi*.

**Figure 4:**
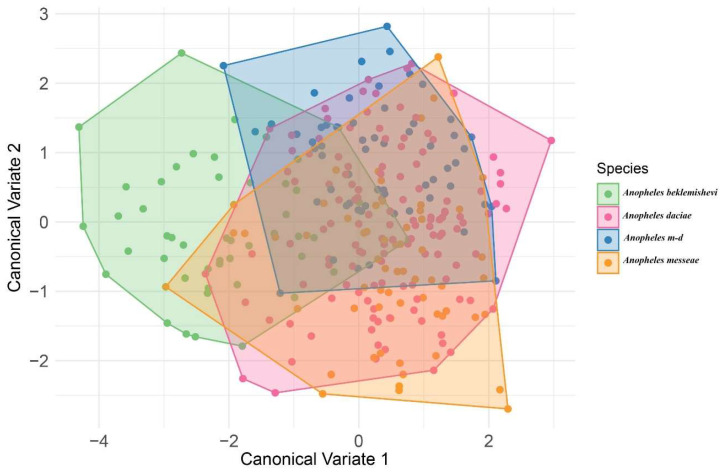
Canonical variate analysis (CVA) of wing shape using species as classifiers. Colors indicate species: green = *An. beklemishevi*; red = *An. daciae*; light blue = hybrids (*An.* m-d); orange = *An. messeae*.

The CVA, used to graphically represent shape variation, showed largely overlapping distributions among species, with only slight displacements between groups (Figure 4). *An. beklemishevi* (green) tended to occupy a more distinct position relative to the others, while hybrids (*An.* m-d, light blue) appeared intermediate, overlapping with both *An. messeae* (orange) and *An. daciae* (red). Although separation was limited, the CVA still illustrated subtle morphological differentiation consistent with interspecific variation. In particular, *An. beklemishevi* displayed the greatest displacement from the other species, whereas *An. daciae* and hybrids overlapped extensively with *An. messeae*.

Procrustes distances confirmed these patterns: the largest distance was observed between *An. daciae* and *An. messeae* (2.5322), whereas the shortest was between *An. daciae* and its hybrids (1.3076), indicating their greater morphological similarity. Although all interspecific distances were statistically significant, the reduced separation between *An. daciae* and *An.* m-d reflects their intermediate wing morphology. Further inspection of wing venation revealed that landmarks located on the radial (R2 + 3) and medial (M1 + 2) veins contributed most consistently to interspecific differences. These landmarks showed stability across localities, supporting their role as reliable morphological markers for species delimitation.

**Figure 5:**
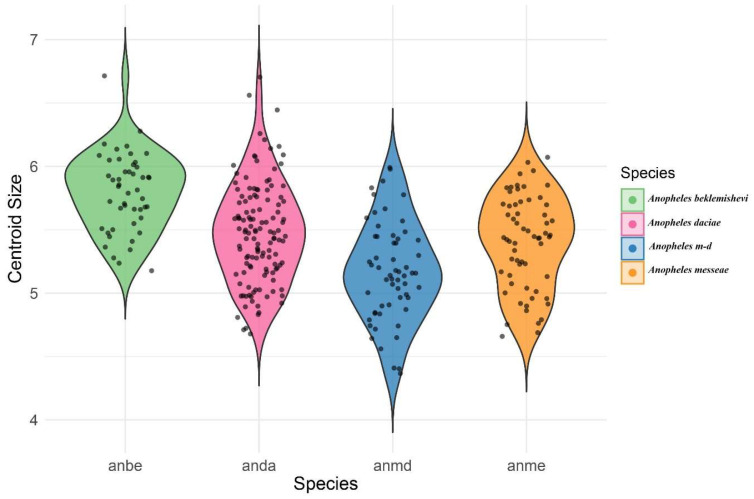
Violin plot showing the distribution of centroid size across the species analyzed.

A violin graph of the distribution of centroid size among species a similar distribution of sizes where the bigger species where *Anopheles beklemishevi* and *Anopheles daciae* (Figure 5).

The Procrustes ANOVA (Table 4) revealed that the difference in CS among species was statistically significant (F = 23.7, *p*-value = 0.001). Additionally, the shape variation among species showed a relatively lower value of F (F = 3.07), but it was still statistically significant (*p*-value = 0.001). The violin plot visualization (Figure 5) clearly illustrates differences in centroid size among species. In this analysis, each species was considered as a population, pooling individuals across sampling localities.

**Figure 6:**
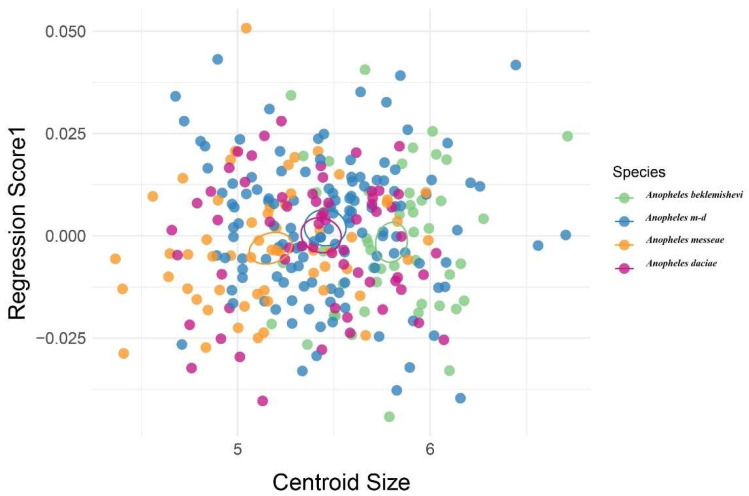
Multivariate regression of wing shape on centroid size in *Anopheles* species. Each dot represents one specimen; colors indicate species (green = *An. beklemishevi*, red = *An. daciae*, light blue = hybrids [*An.* m-d], orange = *An. messeae*) confidence ellipses where calculated using the mean values of every group.

Multivariate regression was used to assess the association between wing shape and centroid size to test for static allometry (Figure 6). The regression showed no significant relationship, indicating that shape variation was not explained by wing size. Morphological differences among species were therefore independent of centroid size. Among the groups, *An. beklemishevi* showed the greatest divergence in shape compared with the other species.

## 4. Discussion

This study provides novel insights into the morphological variation in *Anopheles* mosquitoes in Western Siberia, focusing on wing shape and size through geometric morphometrics (GM). By integrating molecular species identification with morphometric data, we identified clear taxonomic signals that differentiate members of the *maculipennis* subgroup.

Significant differences in centroid size (CS) and wing shape were observed among the four analyzed groups: *An. daciae*, *An. messeae*, *An. beklemishevi*, and *An. messeae* × *An.* daciae hybrids. The largest wing sizes were recorded in *An. beklemishevi* and *An. daciae*, whereas hybrids showed intermediate values and overlapped extensively with their parental species. These patterns are consistent with previous reports in cryptic mosquito groups, where hybrids tend to exhibit intermediate morphologies [17,18,21]. Wing size differences therefore appear to be species-specific, while wing venation traits, particularly those located on radial (R2 + 3) and medial (M1 + 2) veins, provided the most consistent contribution to interspecific differentiation, supporting their reliability as diagnostic landmarks.

The PCA and CVA revealed partial separation among species, with *An. beklemishevi* showing the greatest shape divergence. *An. daciae* and its hybrid *An.* m-d overlapped with *An. messeae*, consistent with their high morphological similarity and the possibility of ongoing gene flow, as previously reported in molecular studies [3,11,20]. This interpretation is further supported by Procrustes distances, where the shortest values were observed between *An. daciae* and *An.* m-d (1.3076), reflecting their intermediate wing morphology. These patterns agree with recent molecular evidence of high hybridization frequencies in some Siberian populations [3].

Environmental conditions across the study sites differed in temperature, breeding season length, precipitation, and effective heat sums (Table 1 and Table 2). In northern taiga habitats such as Berezovo and Bolshaya Sarovka, *An. beklemishevi* and *An. messeae* predominated and exhibited relatively more compact wing shapes, whereas southern forest-steppe and foothill sites such as Kropani and Novoaltaysk were dominated by *An. daciae* and hybrids, which showed more elongated wings. These patterns suggest parallel trends with ecological gradients; however, our data do not allow testing whether they represent ecological responses or species-specific traits.

Previous studies have shown that mosquito wing morphology can be shaped by both intrinsic and extrinsic factors. For instance, *An. funestus* in Cameroon and *An. albimanus* in Colombia exhibited wing variation correlated with climate and geography [17,18], and other morphometric surveys have highlighted the role of environmental gradients [19,20]. Similar trends have also been reported in Asian and European *Anopheles* [19,20,21].

In our dataset, no static allometry was detected (Figure 6), indicating that shape differences were not explained by size. This is consistent with previous reports that wing shape variation among *Anopheles* species is largely independent of centroid size [19,20]. Taken together, these findings emphasize that wing morphology provides reliable taxonomic signals, while the influence of environmental factors remains to be clarified. Although pooling by species across sites facilitated species-level comparisons, this design precludes disentangling ecological influences from intrinsic species differences. Future studies with larger sample sizes and broader geographic coverage will be required to evaluate environmental effects on wing morphology.

## 5. Conclusions

This study provides the first integrative morphometric assessment of Anopheles messeae, *An. daciae*, *An. beklemishevi*, and their hybrids from Western Siberia. Statistically significant differences in wing size and shape were detected among species, with *An. beklemishevi* showing the greatest morphological divergence, while *An. daciae*, *An. messeae*, and hybrids exhibited extensive overlap. The largest wing sizes were recorded in *An. beklemishevi* and *An. daciae*. Venation landmarks on the radial (R2 + 3) and medial (M1 + 2) veins consistently contributed to interspecific separation, underscoring their value as reliable phenotypic markers. Importantly, no static allometry was detected, indicating that wing shape variation was independent of size.

Together, these results demonstrate that wing morphology provides robust taxonomic signals within the *maculipennis* subgroup. The combined use of geometric morphometrics and molecular identification strengthens species delimitation and highlights morphological diversity relevant to the surveillance of malaria vectors in temperate regions.

## Figures and Tables

**Table 1:** Ecogeographic characteristics of sampling sites.

Locality	Subzone	Province	Altitude (m)	Population	Main Water Body
Berezovo	Northern taiga	Severo-Sosvinskaya	31	6411	Vogulka, Ob
Bolshaya Sarovka	Southern taiga	Chulymo-Yeniseyskaya	72	552	Sarovka, Ob
Kolarovo	Birch–aspen forest	Chulymo-Yeniseyskaya	86	376	Tom river
Kropani	Northern forest-steppe	Zauralskaya	84	621	Tobol river, lakes
Novoaltaysk	Piedmont forest-steppe	Verkhneobskaya	140	73,511	Chesnokovka, wetlands
Bystryanka	Mountain forest belt	Verkhneobskaya	222	1838	Katun river

**Table 2:** Seasonal environmental indicators for each site (2021).

Locality	Breeding Season	Duration (Days)	Mean Temp (°C)	Precipitation (mm)	Precip. Rate (mm/Day)	Heat Sum (°C)	Tgtz	Fgtz	Egtz
Berezovo	9 May–5 September	120	13.9	240.9	2.01	545.2	14.9	13	9–10
Bolshaya Sarovka	3 May–28 August	118	14.9	228.3	1.93	624.0	17.1	15	10–11
Kolarovo	3 May–16 September	137	15.5	254.2	1.86	795.3	21.8	19	14–15
Kropani	2 May–16 September	138	18.9	135.2	0.98	1246.2	34.1	30	25–26
Novoaltaysk	27 April–19 September	146	17.1	181.1	1.24	1061.6	29.1	25	20–21
Bystryanka	27 April–19 September	146	17.0	154.4	1.06	1053.6	28.9	25	20–21

**Table 3:** Procrustes ANOVA results for individual variation.

Centroid Size					
Effect	SS	MS	df		*p* (param.)
Individual	93.44285	0.348667	268	8.72	<0.0001
Error 1	10.71869	0.039995	268		
Shape					
Effect	SS	MS	df	F	*p* (param.)
Individual	0.756514	8.3 × 10^−5^	9112	8.57	<0.0001
Error 1	0.088315	9.69 × 10^−6^	9112		

**Table 4:** Procrustes ANOVA results using species and locality as factors.

Centroid Size							
Effect	SS	MS	df	F	*p* (param.)		
Species	10.52242	3.507474	3	23.71	<0.0001		
Locality	1.810702	0.36214	5	2.45	0.0344		
Individual	38.01389	0.147914	257	3.12	0.0003		
Residual	1.375043	0.047415	29				
Shape							
Effect	SS	MS	df	F	*p* (param.)	Pillai tr.	*p* (param.)
Species	0.012278	0.000120372	102	3.07	<0.0001	0.94	<0.0001
Locality	0.008863	5.21366 × 10^−5^	170	1.33	0.0028	0.78	0.0318
Individual	0.342447	3.91905 × 10^−5^	8738	4.38	<0.0001		
Residual	0.008832	8.9578 × 10^−6^	986				

## Data Availability

Data available on demand to the corresponding authors.

## Supplementary Materials

The following supporting information can be downloaded at https://www.mdpi.com/article/10.3390/ani15202949/s1.
