# Supplementary material for: Unraveling Wing Shape Variation in Malaria Mosquitoes from the Arctic Edge: A Geometric Morphometric Study in Western Siberia
Source: Animals (Basel). 2025 Oct 11;15(20):2949. doi: 10.3390/ani15202949 (PMC12560864; doi:10.3390/ani15202949)
Supplement: Supplementary file 1 [file animals-15-02949-s001.zip › animals-3874502-supplementary.pdf]

Table S1. Summary of molecular identification protocol used for *Anopheles* specimens. The PCR-RFLP assay targeted the ITS2 rDNA region using published primers (Artemov et al., 2021). Key reagents, primer sequences, and restriction enzyme information are provided for reproducibility.

Item	Description
Amplified region	ITS2 rDNA
Primers	5,8S_vdir (5′-TGTGAACTGCAGGACACATG-3′), 28S (5′-ATGCTTAAATTTAGGGGGTA-3′)
PCR mixture	1× PCR buffer (16 mM $(NH_4)_2SO_4$; 67 mM Tris-HCl, pH 8.9; 0.1% Tween-20), 2.5 mM $MgCl_2$, 0.2 mM each dNTP, 0.025 U/µL Taq polymerase (Biolabmix, Russia), 0.5 µM each primer
PCR conditions	As described in Artemov et al. (2021)
Restriction enzyme	RsaI (SibEnzime, Russia)
Identification	*An. beklemishevi*: PCR product 771 bp; *An. messeae*: longest restriction fragment 235 bp; *An. daciae*: longest restriction fragment 307 bp
Validation	Subset of 23 samples sequenced (11 *An. messeae*, 7 *An. daciae*, 5 hybrids) as reported in Artemov et al. (2021)
